# Effect of hypnotic suggestion on knee extensor neuromuscular properties in resting and fatigued states

**DOI:** 10.1371/journal.pone.0195437

**Published:** 2018-04-23

**Authors:** Naiandra Dittrich, Daniel Agostino, Roberta Antonini Philippe, Luiz Guilherme A. Guglielmo, Nicolas Place

**Affiliations:** 1 Sports Center, Federal University of Santa Catarina, Physical Effort Laboratory, Florianópolis, Brazil; 2 Institute of Sport Sciences, Faculty of Biology and Medicine, University of Lausanne, Lausanne, Switzerland; 3 Institute of Sport Sciences, Faculty of Social and Political Sciences, University of Lausanne, Lausanne, Switzerland; University of Sydney, AUSTRALIA

## Abstract

**Purpose:**

The aim of this study was to investigate whether hypnotic suggestions can alter knee extensor neuromuscular function at rest and during exercise.

**Methods:**

Thirteen healthy volunteers (8 men and 5 women, 27 ± 3 years old) took part in this counterbalanced, crossover study including two experimental (hypnosis and control) sessions. Knee extensor neuromuscular function was tested before and after hypnosis suggestion by using a combination of voluntary contraction, transcutaneous femoral nerve electrical stimulation and transcranial magnetic stimulation (TMS). A fatiguing exercise (sustained submaximal contraction at 20% maximal voluntary contraction (MVC) force) was also performed to evaluate the potential influence of hypnosis on the extent and origin of neuromuscular adjustments.

**Results:**

Hypnosis did not (p>0.05) alter MVC force or knee extensor neural properties. Corticospinal excitability, assessed with the amplitude of knee extensor motor evoked potentials, was also unchanged (p>0.05), as was the level of intracortical inhibition assessed with paired pulse TMS (short-interval intracortical inhibition, SICI). Time to task failure (~300 s) was not different (p>0.05) between the two sessions; accordingly, hypnosis did not influence neuromuscular adjustments measured during exercise and at task failure (p>0.05).

**Conclusion:**

Hypnotic suggestions did not alter neuromuscular properties of the knee extensor muscles under resting condition or during/after exercise, suggesting that hypnosis-induced improvement in exercise performance and enhanced corticospinal excitability might be limited to highly susceptible participants.

## Introduction

Hypnosis can be defined as an altered state of consciousness in which one person is guided to respond to suggestions aiming at altering perception, sensation, emotion, thought or behavior [1–2]. The efficacy of hypnosis has been well established in the medical field as a pain-reduction intervention [3–4] and also as an anesthesia method before surgery procedures [5–6]. In the field of sport science, hypnosis has been used to reinforce the effects of mental imagery [7] or to overcome the mental discomfort associated with injury [8]. In athletes, the combination of hypnosis and relaxation has been shown to improve precision and thus performance in archery, basketball and golf [9–11].

Some time ago, an interesting study of Ikai and Steinhaus [12] showed an impressive average increase of 25% in the maximum voluntary contraction (MVC) force of the forearm flexor muscles following hypnotic suggestion. These results were in line with previous observations of hypnosis-induced increased strength and endurance observed in upper limb muscles [13]. However, the underlying mechanisms were not identified in those early studies. More recently, Takarada and Nozaki [14] investigated the consequences of hypnotic suggestions on corticospinal excitability by measuring motor evoked potentials (MEP) evoked by transcranial magnetic stimulation (TMS). Contrary to the findings of Ikai and Steinhaus [12], the authors did not find any change in maximal voluntary handgrip force. However, they found enhanced MEP amplitude and increased force exertion when task-motivating suggestions were provided during hypnotic induction. Collectively, these results suggest that hypnosis might alter neuromuscular function and exercise performance.

In the present study, we aimed at investigating whether hypnotic suggestions can alter knee extensor neuromuscular function at rest and during exercise. Specifically, in addition to electrical stimulation of the femoral nerve, we used TMS-induced single and paired stimuli to get insights into potential changes in corticospinal excitability and intracortical inhibition. A sustained isometric knee extension at 20% MVC force was chosen as the exercise model, because task failure in this paradigm has recently been attributed to neural factors [15], which can be modulated by hypnosis. We hypothesized that hypnotic induction would enhance time to task failure of a sustained submaximal isometric contraction through increased corticospinal excitability with no change in MVC force.

## Material and methods

### Participants

Thirteen healthy and physically active subjects (8 men and 5 women, 27 ± 3 years old, 174 ± 9 cm and 70 ± 14 kg) volunteered to participate in this study after having been informed of the experimental procedures and possible risks. The sample size was determined considering the data of Takarada and Nozaki where MEP amplitudes were respectively (mean ± SD) 74±34 and 157±100 μV before and after a task-motivating suggestions following hypnotic induction. The calculated effect size from these data was 1.16, which gave a mimimum sample size of n = 8 (with α level = 0.05 and a power of 90%). We originally aimed for a greater value in case of dropouts and to maximize our chances to detect smaller differences. The study protocol was performed in accordance with the Declaration of Helsinki and approved by the Research Ethics Committees of Vaud canton (protocol 448–15). Before participation, each subject gave written informed consent.

### Experimental protocol

The study involved repeated measures of the same participants. Three trials (at least 72h apart) were performed (one familiarization and two experimental sessions) over a two-week period. Each participant was tested at the same time of day (± 2h) to minimize the effects of biological variation. Participants were asked to refrain from physical activity and caffeine ingestion for a minimum of 24 h prior to testing and instructed to maintain the same meal before each experimental session.

In the first visit, all participants experienced a familiarization session to get used to TMS, voluntary and electrically evoked muscle contractions and to obtain answers to any questions they had regarding the experimental protocol. During familiarization, a hypnosis susceptibility test was also performed for inclusion purpose (see below).

**Fig 1:**
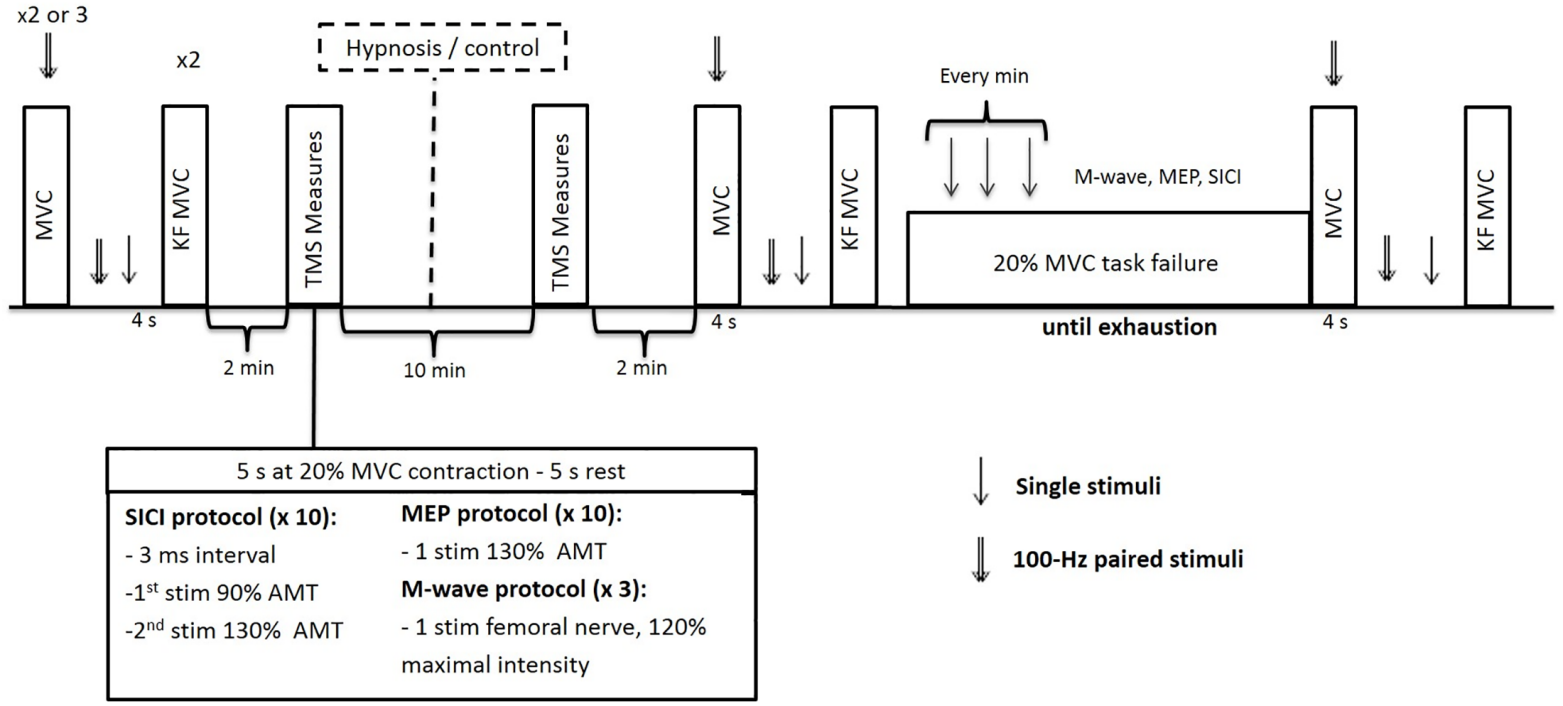
Schematic view of the experimental protocol. MVC = maximal voluntary contraction; KF MVC = Knee flexor maximal voluntary contraction; MEP = motor evoked potential; SICI = short interval intracortical inhibition; AMT = active motor threshold; TMS = transcranial magnetic stimulation.

On separate days, two experimental sessions (counterbalanced order) were performed. Each session began with a warming-up period that included three to six submaximal voluntary isometric knee extensions and flexions between 20% and 80% of the estimated MVC force, followed by a 1 min rest before starting the protocol. The experimental protocol (Fig 1) was as follows: a) two or three MVCs (5% of variation was tolerated between the two last MVCs) of the knee extensors with superimposed doublet (100 Hz), potentiated doublet and a single twitch evoked at every 2 s after the MVCs, separated by 1 min; b) two MVCs of the knee flexors separated by 30 s; c) coil position determination for the TMS; d) active motor threshold (AMT) intensity determination e) two sets of ten 5 s contractions with 5 s recovery and 1 min between the sets at 20% MVC for the measurement of MEP and short-interval intracortical inhibition (SICI); f) three contractions at 20% MVC with a superimposed electrical stimulus on the femoral nerve to obtain the M-wave which will be used to normalize TMS parameters; g) hypnotic induction (hypnosis session) or rest period (control session), duration of about 10 min for both; h) repeat of e) and f) after hypnotic induction or the period of rest; i) one MVC of the knee extensors with superimposed doublet (100 Hz), potentiated doublet and a single twitch evoked at every 2 s after the MVCs; j) one MVC of the knee flexors; k) time to task failure test, which consisted of a sustained isometric voluntary knee extension at 20% MVC until failure, followed (without any interruption of the contraction) by an MVC with superimposed, potentiated doublets and a twitch; l) one MVC of the knee flexors. MEP, SICI and M-wave were also collected every minute (~5 s between each stimulus) during the fatiguing task. The order in which the different methods of stimulation were delivered was kept constant for a given subject and counterbalanced between subjects. During the fatiguing task, each participant received a visual feedback on a computer monitor showing the 20% MVC target force. The contraction was terminated when the subject deviated from the target force for more than 3 consecutive seconds.

### Data collection

#### Evoked contractions

A high-voltage (maximal voltage 400 V) constant-current stimulator (model DS7AH; Digitimer, Hertfordshire, UK) was used to deliver single and paired electrical stimuli to the femoral nerve. Pulse duration was 1 ms, and the interval between paired stimuli was 10 ms. The femoral nerve was stimulated using a circular cathode with a diameter of 5 cm (Dermatrode; American Imex, Irvine, CA) positioned at the femoral triangle level beneath the inguinal ligament. The anode was a 10 x 5-cm rectangular electrode (Compex, Ecublens, Switzerland) fixed on the gluteal fold opposite the cathode. The optimal intensity of stimulation (i.e., that allowing recruitment of all knee extensor motor units) was considered to be reached when an increase in the stimulation intensity did not induce a further increase in the amplitude of the twitch force and M-wave amplitudes of *vastus lateralis* (VL), *vastus medialis* (VM) and *rectus femoris* (RF) muscles. Once the optimal intensity was found, it was further increased by 20% [16].

#### Hypnosis

During the familiarization session, a hypnosis susceptibility test was performed to assess whether the participants were responders to hypnotic suggestions, according to the form C of the Stanford hypnotic susceptibility scale [17]. Moran et al. [18] previously showed a high correlation between susceptibility to hypnosis suggestions and the answer to at least three of the following four elements: eye closure, right hand lowering, right arm rigidity and hands moving together. Thus, for this study, these four elements were considered and the participants who achieved a score ≥ 3 from the 4 elements were included (mean score: 3.3 ± 0.5). One participant (out of 14) was not included in the experimental sessions because he was not susceptible to hypnosis.

After the baseline measurements, hypnotic suggestions were given to the participants by a certified hypnotherapist. In order to induce hypnosis, participants were asked to fix their eyes on a specific point and a chain of suggestions were made. Examples of suggestions include: “My invitation is to sit comfortable in the chair and to focus on your breath, with a recommendation to calm it and to experience a state of internal calm. Try to experience this calm and to focus on your breath”; “I will invite your to reach a place, where you can feel secure, like a safe place. Anytime in the future you wish to reenter this comfortable and pleasant state of calm and relaxation, you will find you can do so just by making yourself comfortable and breathing deeply and slowly for a few minutes, and you will be able to return quickly and easily to this level of deep relaxation and focused awareness whenever you choose.” Participants remained seated on the chair during the hypnotic suggestions, and the experience continued once the hypnotherapist attested that the hypnotic state was reached. This procedure lasted for about 10 min and the hypnotic state was determined assessing the rigidity of the right arm, given the relationship between this parameter ans the depth of the hypnosis state [19]. The hypnotherapist intervened to accompany participants throughout the experiment. For instance, the hypnotic suggestions before MVC included sentences such as: “the force is spread in you”, “you are getting stronger”, “you can perform a maximal voluntary contraction stronger than you did it before”. In addition, the hypnotherapist also guided the subjects during the fatiguing exercise with suggestions such as: “you can maintain this force for longer than that”, “you are strong”, “you can do it”.

#### TMS

Transcranial magnetic stimulation was applied by two Magstim 200^2^ stimulators connected by the Bistim2 module (Magstim, Whitland, Dyfed, UK) as well as a double-cone coil (110 mm mean diameter). All TMS pulses throughout the experiment were administered by the same experienced investigator. TMS was performed over the leg area of the left motor cortex along the nasal-inion axis to induce a postero-anterior current in order to activate the right quadriceps. Surface markings drawn on a swim cap placed on the scalp served as a reference for coil positioning. Optimal coil position was selected so as to elicit the highest average MEP amplitude from VL, VM and RF muscles with a minimal activation of the *biceps femoris* during an active contraction at 20% MVC with an intensity of 50% of maximum stimulator output. Before the delivery of each stimulus, the coil position was verified with regard to the marks on the swim cap. The AMT was defined individually during a contraction at 20% MVC as the minimum stimulus intensity required to evoke visual MEPs in 3 of 6 trials in at least 2 knee extensors. Once AMT was determined, MEPs were elicited and recorded at an intensity of 130% AMT. Paired-pulse measurements were also used to quantify intracortical processes using a sub-threshold conditioning stimulus (90% AMT) followed by a suprathreshold test stimulus (130% AMT) [20–21]. Pulses separated by 3 ms have been shown to produce an inhibitory effect on the test stimulus, known as SICI [22–23].

#### Force recordings

Voluntary and evoked force developed by the knee extensors and flexors was recorded using an isometric ergometer consisting of a custom-built chair equipped with a strain gauge (Universal Load Cell, model 9363-C3, linear range 0–250 N, output sensitivity 2.0 mV•V^−1^, Vishay, Malvern, US). The strain gauge was attached to the chair on one end and securely strapped to the ankle with a custom-made mold. Subjects were seated with a knee angle of 90° and a trunk-thigh angle of 90°. Extraneous movement of the upper body was limited by two crossover shoulder harnesses and a belt across the lower abdomen. In all subjects, the right (dominant) leg was investigated. Force signal was recorded at 1,250 Hz using an AD conversion system (MP150; BIOPAC Systems, Goleta, CA).

#### EMG recordings

The EMG activity of the VL, VM, RF and *biceps femoris* muscles were recorded with pairs of silver chloride (Ag/AgCl) circular (recording diameter = 1 cm) surface electrodes (Medi Trace 100; Kendall, Tyco, Canada) positioned lengthwise over the middle of the muscle belly [according to SENIAM recommendations [24] with an interelectrode (center-to-center) distance of 2 cm. The reference electrode was placed over the patella. Low resistance between the two electrodes (<10 kΩ) was obtained by cleaning and lightly abrading the skin. EMG signals were amplified (gain = 500) with a bandwidth frequency ranging from 10 to 500 Hz, digitized at a sampling frequency of 5,000 Hz, and recorded by the AD conversion system. Isometric force and EMG data were stored and analyzed offline with commercially available software (AcqKnowledge software; BIOPAC Systems, Goleta, CA).

#### RPE

Rating of Perceived Exertion (RPE) was assessed at every ~30s using the 6–20 Borg scale [25].

### Data analysis

#### Force data

MVC and evoked forces were considered as the peak force attained during the contraction. The maximal voluntary activation level (VA%) during MVCs was estimated with the twitch interpolation technique according to the following formula: VA% = [1- (superimposed doublet force x force level at stimulation / MVC force) / potentiated doublet force] x 100 [26].

#### EMG data

EMG signals during the MVCs were quantified as root mean square (RMS) amplitude for a 500-ms interval around maximum force (250-ms periods either side of the peak force). M-wave peak-to-peak amplitude from VL, VM and RF muscles was measured from the single stimulation. EMG RMS of VL, VM and RF muscles were also quantified for three seconds at five different points during exercise (0%, 25%, 50%, 75% and 100% of time to task failure). These RMS values were normalized by the maximal RMS obtained during MVC before exercise. In addition, the RMS values obtained during MVC were normalized by the respective M-wave amplitudes (RMS/M) to get another index of central activation. MEPs from VL, VM and RF muscles were measured as the peak-to-peak amplitude response; for each set the average of 10 was used and normalized by the average amplitude of the three M-wave elicited at the same force level at the corresponding time point. The average of the 10 SICI responses was expressed as a percentage of the mean MEP amplitude elicited by the single TMS pulse. During exercise, MEP and SICI are presented at 0%, 50% and 100% of the time to task failure. Due to technical problems, MEP and SICI data could only be obtained in 11 participants during exercise.

#### RPE

RPE data were analyzed at 25%, 50%, 75% and 100% of the time to task failure.

### Statistical analysis

Data normality was checked with the Shapiro–Wilk test. A two-way repeated measures analysis of variance (ANOVA) was used to examine the differences between conditions (hypnosis vs. control) over time (pre vs. post or relative percentage of time to task failure). Post hoc analyses (Tuckey procedure) were used to test for differences among pairs of means when appropriate. SigmaPlot software for Windows (version 11; Systat, Chicago, IL) was used for the statistical analyses. Data are presented as mean ± standard deviation. The level of significance was set at p<0.05.

## Results

### Effect of hypnosis on neuromuscular function at rest

**Fig 2:**
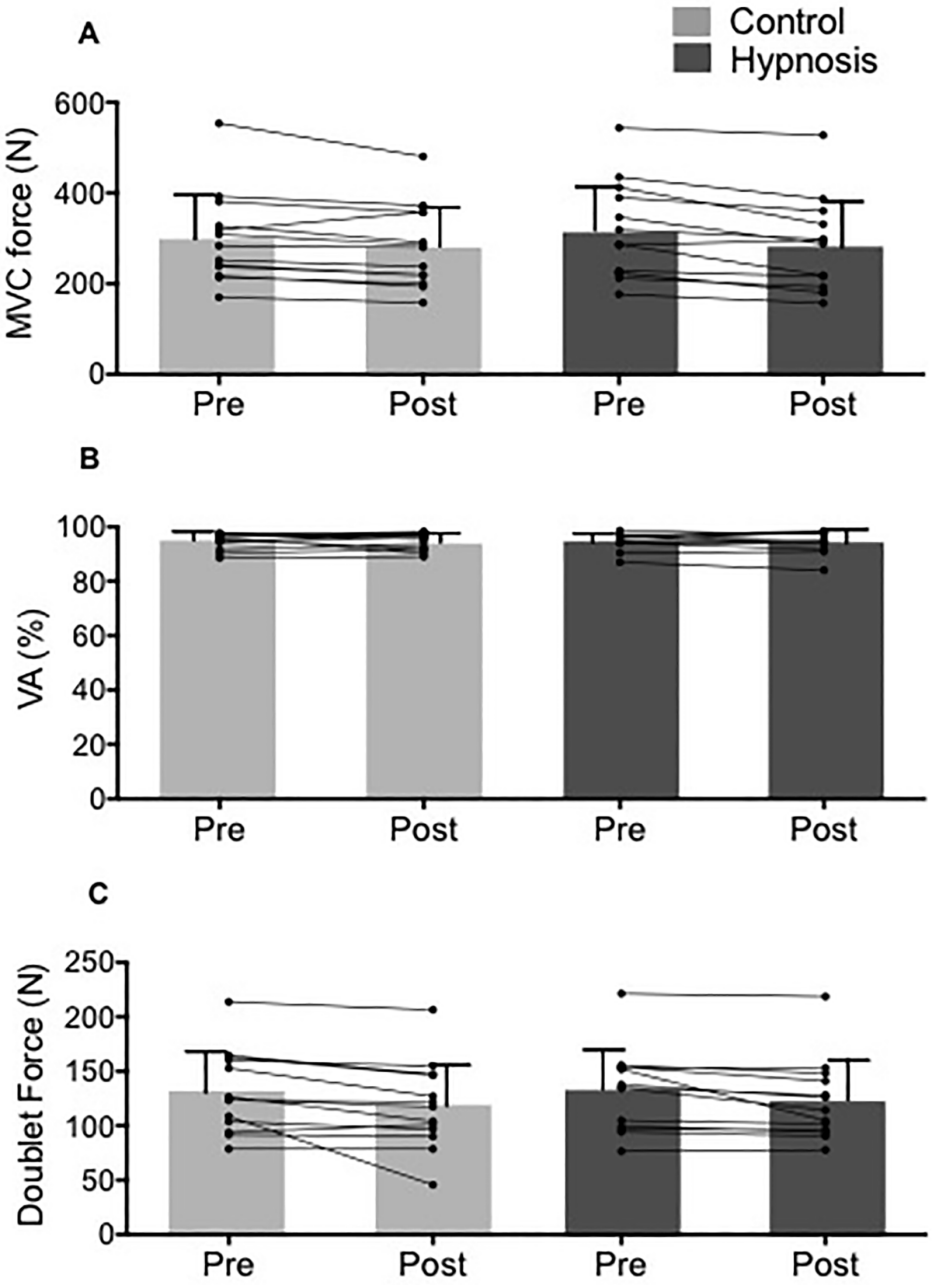
Hypnosis did not influence knee extensor neuromuscular properties. A. Maximal voluntary contraction (MVC) force; B. Maximal voluntary activation level (VA%) and C. Peak doublet force measured before and after control/hypnosis suggestion. Pre = baseline measurements; post = post hypnosis/control; * p<0.05 compared to Pre. Data are presented as mean ± SD and circles represent individuals values.

As presented in Fig 2A, hypnosis had no impact on MVC force. However, MVC force was slightly depressed after the 10 min period of hypnotic induction or rest (control -6.2 ± 3.2%; hypnosis -10.8 ± 0.6%, p<0.05). VA% and doublet force were unchanged (p>0.05) after this period (Fig 2B and 2C). Peak twitch was also unchanged (p>0.05) in both experimental sessions (hypnosis = 97 ± 26 N vs. 88 ± 22 N; control = 95 ± 25 N vs. 90 ± 26 N). RMS/M values and M-wave amplitude from VL, VM and RF muscles (data not shown for sake of clarity) were unchanged (p>0.05) after hypnosis suggestion or the resting period.

**Fig 3:**
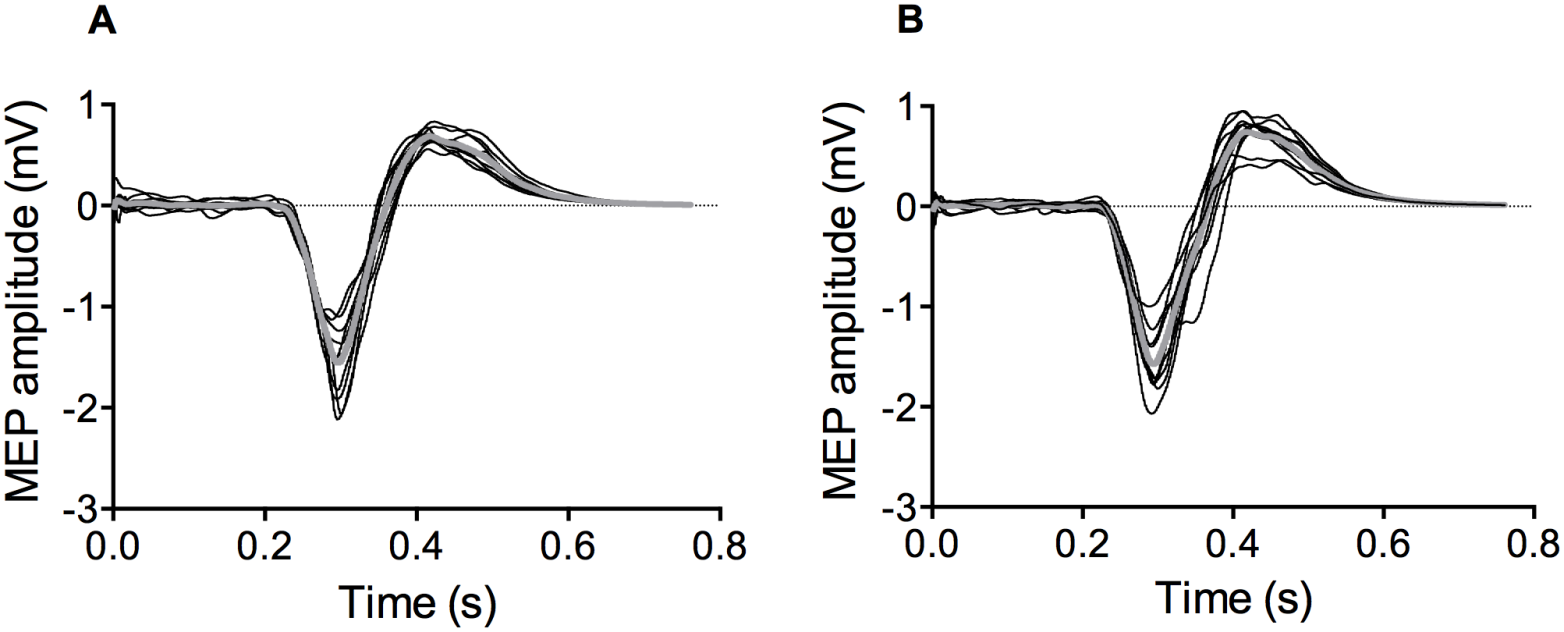
Original MEPs recordings from the vastus lateralis muscle. Raw traces are presented for one representative subject with the thick line representing the average of the 10 MEP (thin lines) measured before (A) and after (B) hypnotic suggestion.

Fig 3 shows original recording of MEP from the VL muscle measured before and after hypnosis suggestion. As can be seen from the figure, corticospinal excitability of the knee extensor muscles remained unchanged after hypnosis.

No significant difference in AMT were found between both sessions (30 ± 4% vs. 31 ± 3% maximal stimulator output for hypnosis and control sessions, respectively (p>0.05). Before exercise, hypnosis had no influence on knee extensor MEP and SICI (Table 1).

**Table 1:** Normalized values of MEP and SICI parameters (mean ± SD) obtained before and after hypnosis induction / resting period. MEP amplitude was normalized by M-wave amplitude and SICI amplitude was normalized by MEP amplitude. There was no significant difference after the 10-min rest period (control) or hypnosis.

Variable	Control		Hypnosis	
	Pre	Post	Pre	Post
MEP_VL_	0.27 ± 0.14	0.31 ± 0.14	0.25 ± 0.07	0.24 ± 0.09
MEP_VM_	0.46 ± 0.58	0.44 ± 0.41	0.28 ± 0.11	0.26 ± 0.10
MEP_RF_	0.34 ± 0.12	0.39 ± 0.20	0.36 ± 0.20	0.33 ± 0.21
SICI_VL_	0.30 ± 0.14	0.28 ± 0.13	0.29 ± 0.14	0.31 ± 0.15
SICI_VM_	0.26 ± 0.11	0.23 ± 0.09	0.24 ± 0.11	0.27 ± 0.15
SICI_RF_	0.24 ± 0.07	0.20 ± 0.07	0.20 ± 0.07	0.26 ± 0.18

MEP = motor evoked potential, SICI = short interval intracortical inhibition. VL = *vastus lateralis*, VM = *vastus medialis*, RF = *rectus femoris*.

### Effect of hypnosis on neuromuscular fatigue

**Fig 4:**
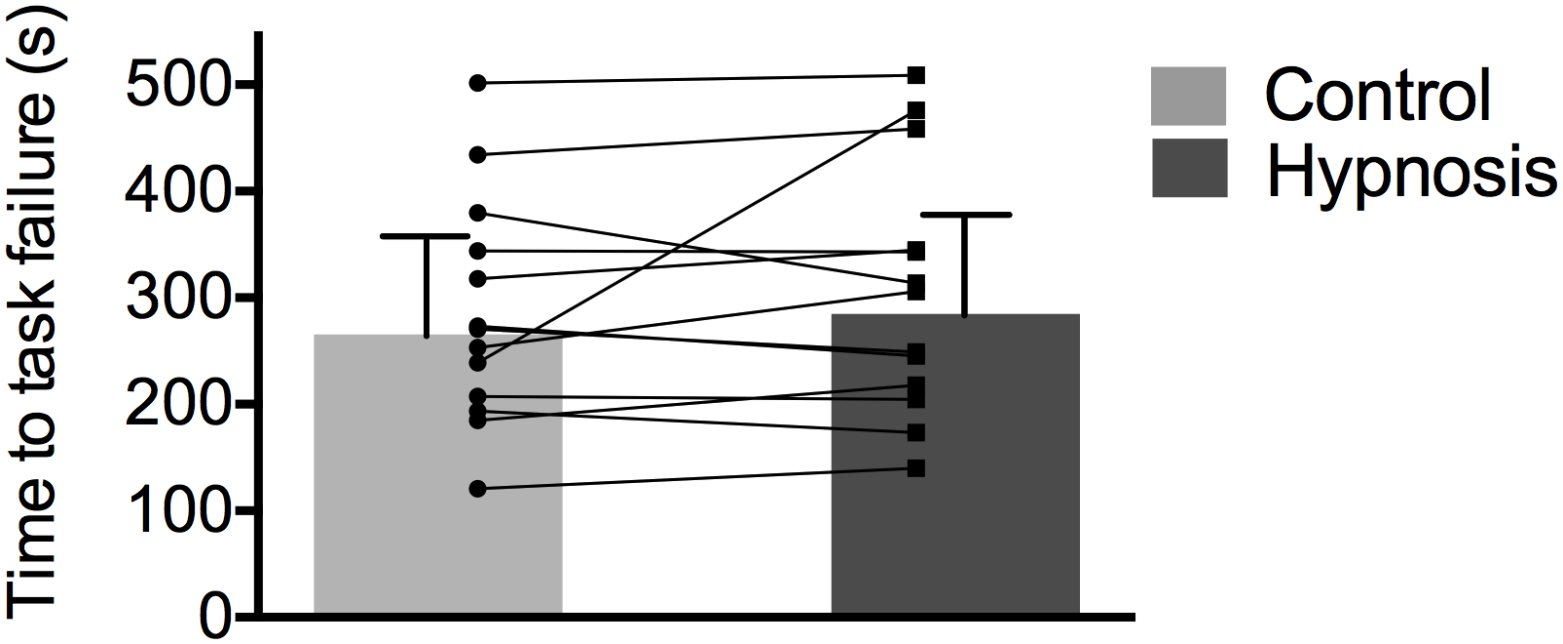
Time to task failure was similar for both sessions. Data are presented as mean **±** SD and circles represent individuals values.

There was no significant difference in the time to task failure between hypnosis and control conditions (hypnosis = 306 ± 118 s; control = 286 ± 107 s, p> 0.05, Fig 4).

**Fig 5:**
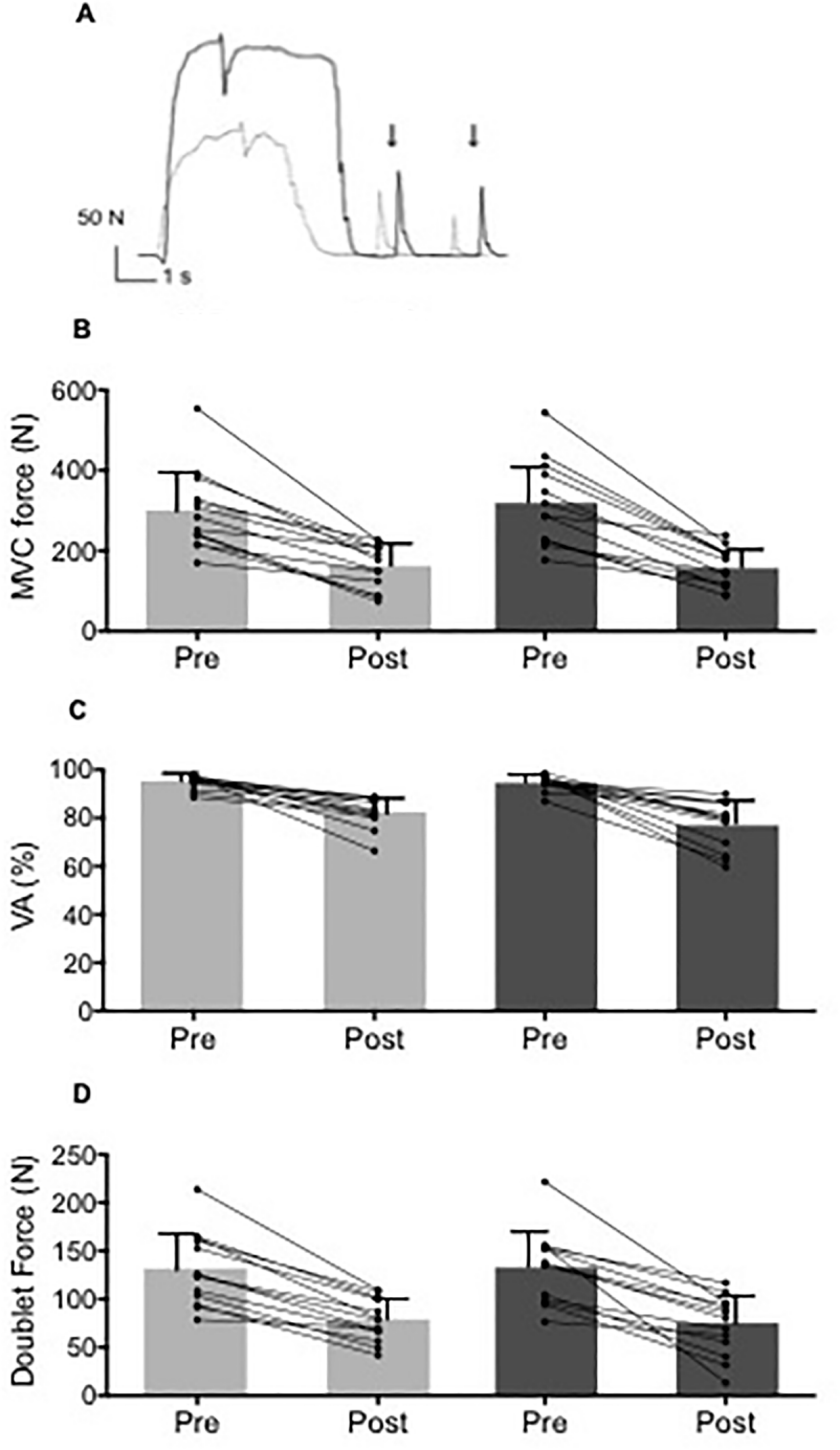
The extent and origin of neuromuscular fatigue was similar in both sessions. A. Original recording of an MVC with a superimposed doublet and a potentiated doublet and single twitch measured before and after time to task failure in the hypnosis session. B. Maximal voluntary contraction (MVC) force. C. Maximal voluntary activation level (VA%); D. Peak doublet force measured before and after endurance time in both conditions. *p<0.05 compared to Pre. Panels B-D are mean **±** SD and circles represent individuals values.

Exercise induced a similar decrease in MVC force (hypnosis = -49 ± 11%; control = -44 ± 14%), VA% (hypnosis = -17 ± 9%; control = -13 ± 7%) and doublet force (hypnosis = -35 ± 16%; control = -36 ± 13%) in each session (Fig 5). Knee flexor MVC force was unchanged in both experimental sessions (hypnosis = 79 ± 27 N vs. 75 ± 30 N; control = 90 ± 40 N vs. 77 ± 31 N respectively before and after exercise, p>0.05).

There was no difference between both sessions for M-wave amplitude, which remained stable after exercise for VL and RF muscles, whereas a slight decrease (~5–10%, p<0.05) was observed in VM (Table 2).

**Table 2:** M-wave peak to peak amplitude (mean ± SD) from *vastus lateralis* (VL), *vastus medialis* (VM) and *rectus femoris* (RF) muscles measured before and after exercise.

	Control		Hypnosis	
	Pre	Post	Pre	Post
VL	14.7 ± 7.9	14.5 ± 7.5	12.2 ± 7.9	10.9 ± 7.4
VM	15.1 ± 7.2	14.4 ± 6.6*	14.8 ± 7.2	12.9 ± 6.6*
RF	9.7 ± 5.6	7.9 ± 3.6	6.9 ± 5.6	6.1 ± 3.6

Pre = measurements made before hypnosis/control; post = post task failure. VL = *vastus lateralis*, VM = *vastus medialis*, RF = *rectus femoris*.

*p < 0.05 compared to Pre.

**Fig 6:**
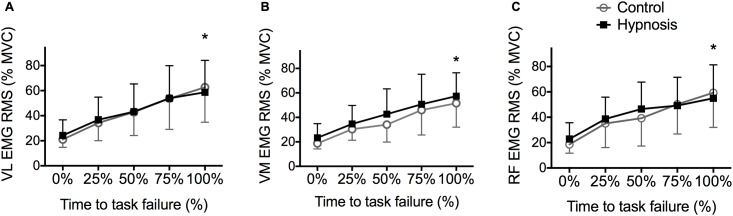
The increase in EMG activity during exercise was not influenced by hypnosis. RMS EMG activity measured during exercise, and normalized to the maximum RMS EMG obtained during the MVC performed before exercise, from: A. *vastus lateralis* (VL), B. *vastus medialis* (VM) and C. *rectus femoris* (RF) muscles. * p<0.05 compared to 0% and 25% of the time to task failure. Data are presented as mean ± SD.

Fig 6 shows that EMG activity increased similarly for the three knee extensors during exercise (from ~20 to ~50% maximal EMG, p<0.05), and there was no difference between sessions (p>0.05).

**Fig 7:**
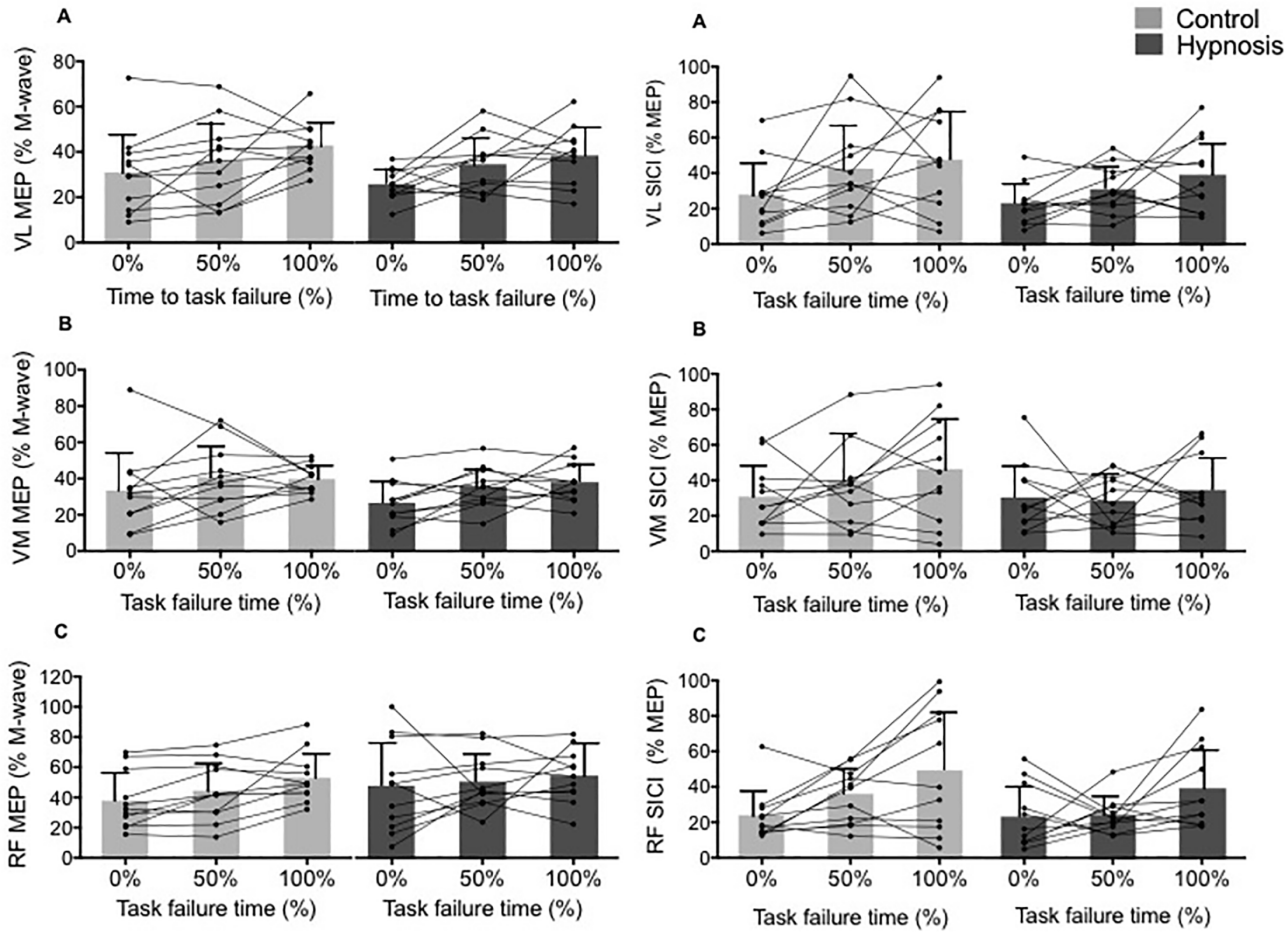
MEP and SICI values increased during exercise with no influence of hypnosis. Normalized MEP and SICI measured at 0, 50 and 100% of the time to task failure. MEP = motor evoked potential, SICI = short interval intracortical inhibition. VL = *vastus lateralis*, VM = *vastus medialis*, RF = *rectus femoris*. * p<0.05 compared to the start of exercise (0%). Data are presented as mean ± SD and circles represent individuals values.

There was no effect of hypnosis on MEP and SICI parameters measured during exercise. However, most of these parameters showed increased values (p<0.05) towards the end of exercise (Fig 7).

**Fig 8:**
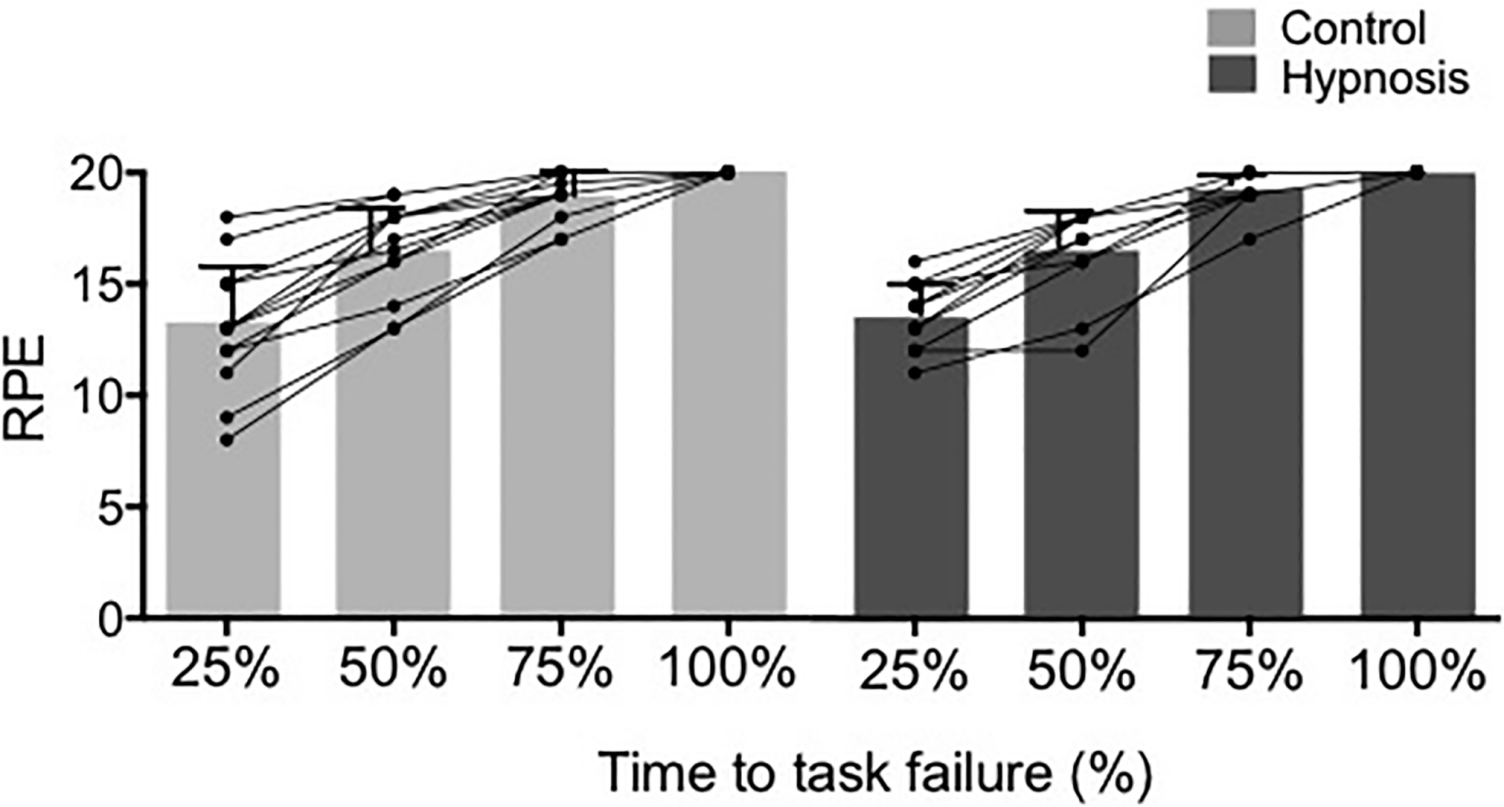
The increase in the rate of perceived exertion (RPE) during exercise was similar for both sessions. * p<0.05 compared to 25% of the time to task failure. Data are presented as mean ± SD and circles represent individuals values.

RPE increased during exercise, but no difference was noted between conditions (Fig 8).

## Discussion

The present study was designed to test the hypothesis that hypnotic suggestions would increase knee extensor corticospinal excitability and, therefore, increase time to task failure of a sustained isometric contraction. Contrary to our hypothesis, we found no effect of hypnosis on knee extensor neuromuscular function at rest and during exercise. In agreement with these findings, time to task failure was similar for both sessions.

### No effect of hypnosis on knee extensor neuromuscular function at rest

A pioneer study of Ikai and Steinhaus [12] showed an increase (~25%) in MVC force under hypnosis. This impressive improvement was attributed to the fact that hypnosis was able to remove inhibitory influences [13]. Our data showed no increase in knee extensor MVC and confirm more recent results from Takarada and Nozaki [14] for handgrip force. Although no change was reported for MVC force, potential neural adaptations induced by hypnosis could not be discarded. Here we assessed the mechanisms of the potential neural adaptations with electrical nerve stimulation and transcranial magnetic stimulation. Our results indicated no effect of hypnosis on the extent of descending drive to the muscle, as both %VA and RMS/M remained unchanged after hypnotic suggestion. In addition, according to our expectations, we found no effect of hypnosis suggestions on peripheral properties such as neuromuscular propagation (M-wave amplitude) and contractility (evoked forces).

Contrary to our findings, Takarada and Nozaki [14] reported a significant increase in MEP amplitude (~ twofold)) when a task-motivating suggestion was provided during hypnotic induction. One of the reasons for this inconsistency could be related to differences in participants’ susceptibility to hypnosis since in Takarada and Nozaki [14] only highly susceptible subjects were included. Indeed, while we included only participants with a score ≥ 3 out of 4 items from the Standford Hypnotic Susceptibility Scale-Form C, Takarada and Nozaki [14] included participants with a score of 8 out of 12 using the same scale. In addition, these authors used the resting motor threshold to determine the stimulus intensity to evaluate corticospinal excitability. However, previous studies showed that resting motor threshold might require high stimulus intensities especially with the lower limbs, and that it can even sometimes not be determined due to a lower excitability in the resting state [27]. High-intensity stimulus using resting motor threshold can also induce a contraction of both agonist and antagonist muscles, whereas the use of an active muscle may reduce the variability between stimuli because it stabilizes corticospinal excitability, attention and somatosensory influences [28]. Noteworthy, our normalized MEP values are consistent with the recent work of O’leary et al. [28] measured on the active VL muscle (10% MVC force).

The effects of hypnosis are presumably mediated through its effects on brain activation as evidenced by some studies that have used imaging technique. For example, Hoeft et al [29], using magnetic resonance imaging (fMRI), showed that hypnosis can modulate the functional connectivity between various brain areas. Crawford et al. [30] observed using electroencephalogram that under hypnosis subjects presented a higher activity of theta waves (frequency band between 4 to 7 Hz), and a decreased activity of alpha waves (frequency band between 8 and 13 Hz), which is related to mental relaxation [31]. In our study, we measured SICI as an index of intracortical inhibition. Previous studies showed that SICI is related to changes in GABAergic cortical inhibitory interneuron activity [32] and its evaluation can provide information regarding the balance between excitatory and inhibitory circuits [33]. Only few studies have examined SICI on the knee extensors and a good reliability was recently reported for both MEP and SICI measured on the VL muscle by using a comparable approach to ours to localize the stimulation point [28]. However, a large variability has been observed between studies, with SICI values ranging from 40 to 90% of the MEP values [20, 28, 34–37]. Here we used 90% AMT as it was previously shown that the magnitude of SICI (i.e. low SICI/MEP ratio) was optimal at this intensity [20,28]. This discrepancy in the literature is certainly explained by the condition (active / resting muscle) and the stimulation intensities used for both the conditioning and the test stimulus. Consistent with our findings on MEP values, our results did not show any alteration in the SICI values, suggesting that hypnosis did not alter the level of intracortical inhibition.

### No effect of hypnosis on knee extensor neuromuscular function during exercise

Time to task failure of a sustained submaximal contraction is highly associated to the ability of the central nervous system to maintain a sufficient activation of the exercised muscles [15, 38–39]. Thus, considering that hypnosis might lead to a better preservation of neural drive to the muscle during exercise, we also investigated the potential influence of hypnosis suggestions on the origin and extent of neuromuscular fatigue. Results showed a time to task failure of ~300 s, which is in agreement with our previous studies [15,40]. However, hypnosis had no influence on time to task failure. In line with this result, knee extensor EMG activity increased similarly during exercise in both sessions. As previously observed [15,40], EMG activity at task failure was much lower than maximal EMG (~50% maximal EMG measured before exercise). In addition, participants were able to develop on average ~50% MVC force when asked to perform a brief MVC but were not able to sustain the 20% MVC force level any more. These two observations clearly indicate that neural factors were involved in the process of task failure but hypnosis failed to improve performance. Accordingly, the increase in RPE during exercise was not influenced by hypnosis. This results contrast with previous findings showing that hypnosis may alter RPE by changes in the brain activation pattern related to the sense of effort in participants highly susceptible to hypnosis [41]. It should also be noted that a few participants reported difficulty to keep concentrated on hypnosis suggestions during exercise while maintaining the eyes open to focus on the target force.

In addition to the central fatigue evidenced by the reduction in %VA during post exercise MVC, we reported large alterations in evoked force (-35% for the doublet) with minimal change for the knee extensor M-wave amplitude. Therefore, peripheral fatigue was more likely to originate from intramuscular impairment (involving Ca^2+^ handling, Ca^2+^ sensitivity and/or myofibrillar function) than neuromuscular propagation as previously proposed using the same exercise model [15].

We also investigated corticospinal excitability and intracortical inhibition during exercise and again found no influence of hypnosis suggestions, as reported under resting condition. However, we reported a significant increase in MEP and SICI/MEP ratio towards the end of exercise, which can be explained by increased motor unit recruitment to counteract the effect of fatigue and maintain the required force level [42]. The increased corticospinal excitability reflected by the increase in MEP amplitude is consistent with previous studies having used the model of sustained submaximal contraction [42–43]. It has also been suggested that the increase in the SICI/MEP ratio represents a compensatory effect to ensure adequate cortical excitation in response to the fatigue-induced reduction in muscle force [43].

### Conclusion

Hypnotic suggestions did not alter neuromuscular properties of the knee extensor muscles, as evaluated by the combination of voluntary contraction, femoral nerve stimulation and transcranial magnetic stimulation. Corticospinal excitability increased during exercise, presumably due to a reduction in intracortical inhibition, which was not influenced by hypnosis. As a consequence, both time to task failure and the origin and extent of neuromuscular fatigue were similar with or without hypnosis. Our results therefore suggest that hypnosis-induced improvement in exercise performance and enhanced corticospinal excitability might be limited to highly susceptible participants.
